# Breast-conserving surgery is an appropriate procedure for centrally located breast cancer: a population-based retrospective cohort study

**DOI:** 10.1186/s12893-023-02181-6

**Published:** 2023-10-03

**Authors:** Ye-Wei Yuan, Peng-Cheng Liu, Fang-Fang Li, Ya-Han Yang, Wei Yang, Li Fan, De-Wu Mou, Hong-Wei Yang, Mao-Shan Chen

**Affiliations:** 1 Department of Breast and Thyroid Surgery, Sichuan Provincial Hospital for Women and Children (Affiliated Women and Children’s Hospital of Chengdu Medical College), Chengdu, 610041 People’s Republic of China; 2 Department of Burn and Plastic Surgery, West China Hospital, Sichuan University, 37 Guoxue Street, Chengdu, 610041 People’s Republic of China; 3 Department of Operating Room, Suining Central Hospital, 127 Desheng Road West, Suining, 629000 People’s Republic of China; 4 Medical Imaging, North Sichuan Medical College, Nanchong, 637000 People’s Republic of China; 5 Sichuan Center for Disease Control and Prevention, Chengdu, 610041 People’s Republic of China; 6 Department of Breast and Thyroid Surgery, Suining Central Hospital, 127 Desheng West Road, Suining, 629000 People’s Republic of China

**Keywords:** Breast cancer, Tumor location, Breast-conserving surgery, Mastectomy, Survival, SEER program

## Abstract

**Background:**

The evidence of breast-conserving therapy (BCT) applied in centrally located breast cancer (CLBC) is absent. This study aims to investigate the long-term survival of breast-conserving therapy (BCT) in centrally located breast cancer (CLBC) compared with mastectomy in CLBC and BCT in non-CLBC.

**Methods:**

Two hundred ten thousand four hundred nine women with unilateral T1-2 breast cancer undergoing BCT or mastectomy were identified from the Surveillance, Epidemiology, and End Results database. Kaplan–Meier survival curves were assessed via log-rank test. Propensity score matching (PSM) was used to balance baseline features, and the multivariable Cox model was used to estimate the adjusted hazard ratio [HR] and its 95% confidence interval [CI] for breast cancer-specific survival (BCSS) and overall survival (OS).

**Results:**

With a median follow-up of 91 months, the BCSS and OS rates in patients who received BCT were greater than those patients treated with mastectomy in the entire CLBC set. Multivariable Cox analyses showed that CLBC patients who received BCT had better BCSS (HR = 0.67, 95%CI: 0.55–0.80, *p* < 0.001) and OS (HR = 0.78, 95%CI: 0.68–0.90, *p* = 0.001) than patients who received a mastectomy, but there were no significant differences of BCSS (HR = 0.65, 95%CI: 0.47–0.90, *p* = 0.009) and OS (HR = 0.82, 95%CI: 0.65–1.04, *p* = 0.110) after PSM. In patients treated with BCT, CLBC patients had a similar BCSS (HR = 0.99, 95%CI: 0.87–1.12, *p* = 0.850) but a worse OS (HR = 1.09, 95%CI: 1.01–1.18, *p* = 0.040) compared to that of the non-CLBC patient, but there was no significant difference both BCSS (HR = 1.05, 95%CI: 0.88–1.24, *p* = 0.614) and OS (HR = 1.08, 95%CI: 0.97–1.20, *p* = 0.168) after PSM.

**Conclusion:**

Our findings revealed that BCT should be an acceptable and preferable alternative to mastectomy for well-selected patients with CLBC.

**Supplementary Information:**

The online version contains supplementary material available at 10.1186/s12893-023-02181-6.

## Introduction

Centrally located breast cancer (CLBC) refers to the location of cancer in the nipple areola or the central region of the breast. Although breast-conserving treatment (BCT) has become the standard care for early-stage breast cancer, direct evidence of BCT for CLBC patients is lacking [1, 2].

BCT consists of breast-conserving surgery (BCS) and radiotherapy, which has been proven to be at least equivalent or even superior to mastectomy concerning survival outcomes [3–5]. The choice between BCS and mastectomy also depends on the tumor’s location, marginal status, adjuvant therapy, and cosmetic appearance [6, 7]. The patient’s willingness and the recurrence risk are important factors influencing the choice of BCS [6, 7].

CLBC had different clinical features compared with non-CLBC. Patients with CLBC had a higher axillary lymph node metastasis rate, higher possibility of positive margin and invasion of the NAC, lower satisfying cosmetic outcome, and increased local recurrence rate [8, 9]. The published data regarding BCS in CLBC are scarce; only limited studies with a small sample size support the safety of BCS in CLBC [10–13]. Several researches based on real-world data concluded that BCS may be an alternative for CLBC patients. However, these studies had limitations in the study design, including advanced breast cancer, absence of post-surgical radiotherapy, small sample, and insufficient follow-up, which cannot provide evidence for the utility of BCT in patients with early-stage CLBC [14–19]. Further study is needed to evaluate the long-term oncological safety of BCT in CLBC.

Limited evidence supports BCS as a safe oncological alternative to mastectomy in CLBC patients. Hence, we hypothesized that the prognosis of BCT in CLBC is like that of mastectomy in CLBC, and like that of BCT in non-CLBC patients. We conducted a retrospective cohort study using the data extracted from the US Surveillance, Epidemiology, and End Results (SEER) database, to analyze the overall and cancer-specific survival between BCT and mastectomy in CLBC patients, and between CLBC and non-CLBC patients treated with BCT.

## Methods

We used the data identified from the SEER database, which represents approximately 30% of the American population (https://seer.cancer.gov/). Informed consent was not required because personal identifying information was not accessed and no intervention was conducted. This study protocol was approved by the Clinical Research Ethics Committee of the Suining Central Hospital (No. LLSLH20220013). This study was conducted and reported according to the Strengthening the Reporting of Observational Studies in Epidemiology (STROBE) statement [20].

### Patients selection

Data were retrieved using the SEER*Stat version 8.3.9 on May 23, 2022 (user name: 10143-Nov2021). The case list of breast cancer who met the following inclusion criteria was generated: aged 18 to 70-year-old; female, year of diagnosis between 2004 and 2015, histologically diagnosed as breast carcinoma, breast cancer was the first primary carcinoma, tumor stage was T1-2, N0-3 and M0, local surgery was performed. Patients with one of the following exclusion criteria were excluded: tumor accumulation in the whole breast or unknown tumor location, surgery procedure unknown, without radiotherapy after BCS, without follow-up data, or tumor stage.

Patients were classified as CLBC group if the case with a tumor located in the nipple (code: C500) or central portion of the breast (code: C501). In contrast, the remaining patients were classified as the non-CLBC group. Then, patients were divided into BCT (including BCS and radiotherapy) and mastectomy cohorts according to the breast surgical procedure (Supplementary Table 1).

### Variables

The sociodemographic features, clinicopathological characteristics, and survival data were extracted from the database. According to the age at diagnosis, patients were divided into groups of 18–40 years, 41–50 years, 51–60 years, and 61–70 years. Marital status was classified as unmarred (including single, separated, unmarried or domestic partner, widowed, and divorced), married, and unknown. The histological type was classified into three subtypes: invasive ducal carcinoma (IDC, code:8500/3), invasive lobular carcinoma (ILC, code: 8520/3), and others according to the 3^rd^ edition of the International Classification of Diseases for Oncology (ICD-O-3). Tumor TNM stage was classified referenced to the standard of the 6^th^ edition of the breast cancer anatomical staging system of the American Joint Committee on Cancer (AJCC) [21]. Estrogen receptor (ER) status and progesterone receptor (PR) status were divided into negative, positive, and unknown. The human epidermal growth factor receptor-2 (HER-2) status was available from 2010, which was classified as negative, positive, and unknown. The breast surgery was classified into the BCS group (code: 19 and 20) and the mastectomy group (code: 30–76, 80).

The primary outcomes of this study were breast cancer-specific survival (BCSS) and overall survival (OS). BCSS was calculated from the date of breast cancer diagnosis to the date of death from breast cancer or the last follow-up for patients still alive. OS was computed from the time of diagnosis until the time of death from any cause, or the last follow-up for patients still alive.

### Statistical analysis

All variables were converted to categorical variables and presented with percentages. The Chi-square test was used to compare patient-specific variables between BCT and mastectomy in the CLBC cohort and between CLBC and non-CLBC in the BCT cohort. To overcome the effects of baseline differences on survival outcomes, the propensity score match (PSM) method was used to balance baseline features between BCT and mastectomy patients in the CLBC cohort, and between CLBC and non-CLBC patients in the BCT cohort at a ratio of 1:1, respectively. Survival outcomes were estimated using the Kaplan–Meier plot and compared across groups using the log-rank test. Univariable and multivariable Cox proportion risk regression models were used to identify the prognostic factors for BCSS and OS, and hazard ratios (*HR*s) with 95% confidence intervals (*CI*s) were calculated. Variables with *p* < 0.1 in univariable analysis or with a clinical consideration were enrolled in the multivariable model. Statistical analyses were performed by R software (version 4.0.3 for Windows) using the packages “survival” and “survminer”. All statistical tests were two-sided, and *p* < 0.05 was considered statistically significant.

## Results

### Baseline characteristics

**Fig. 1:**
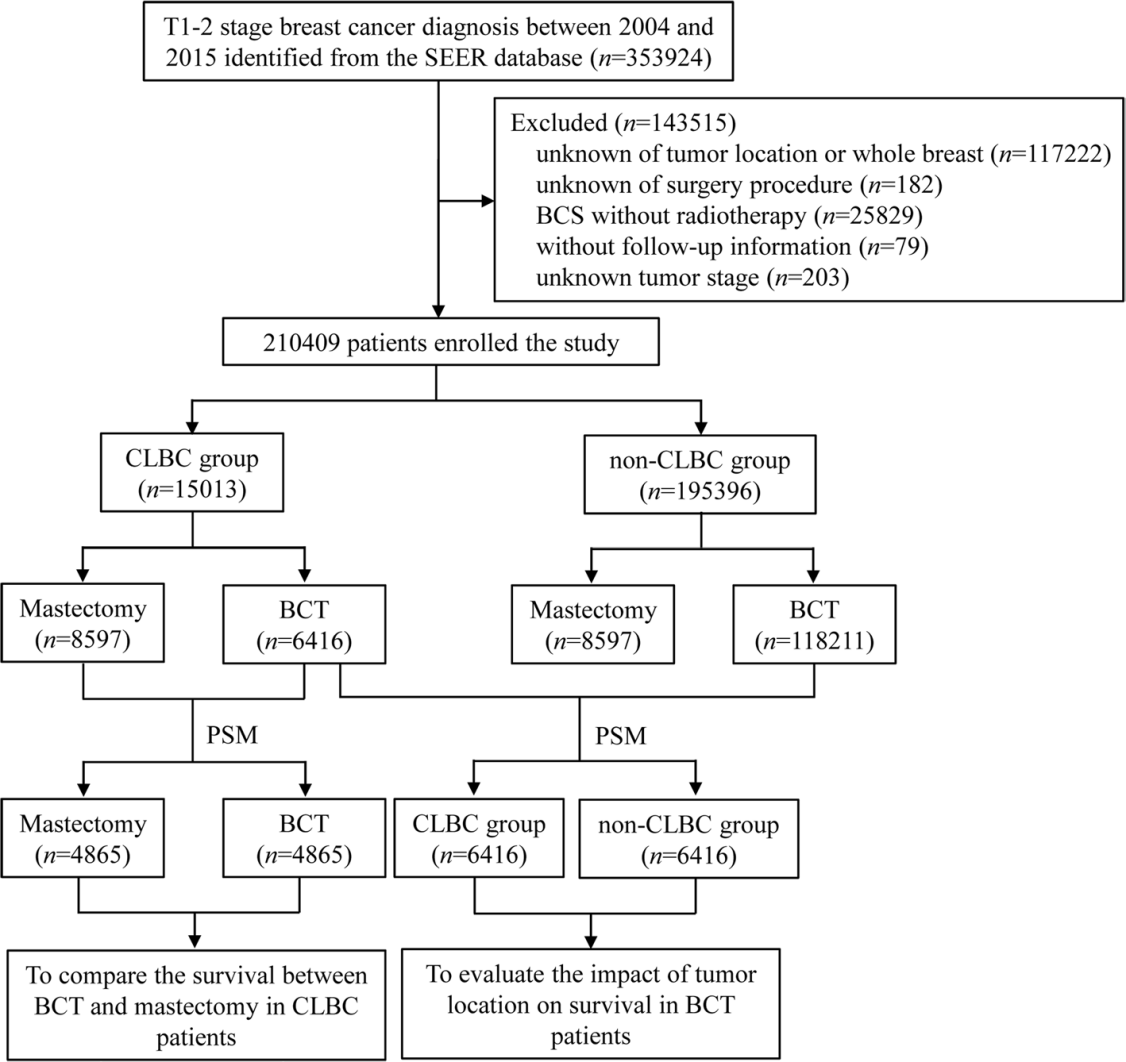
Study flow chart and patient selection

**Fig. 2:**
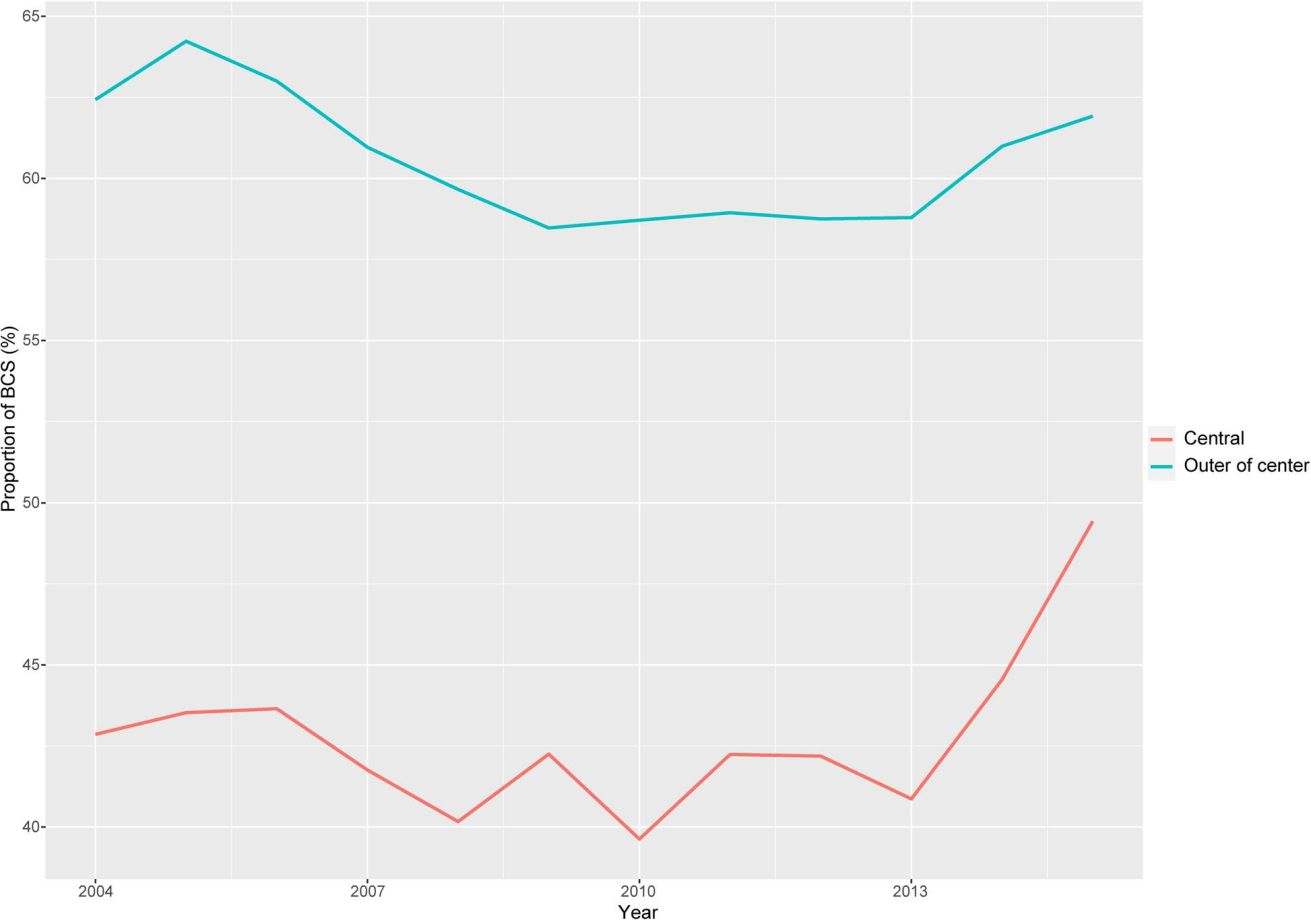
Trends of BCS rate from 2004 to 2015 in patients with CLBC and non-CLBC

Finally, 210,409 patients with stage T_1-2_ non-metastatic breast cancer as their first malignancy were retrieved (Fig. 1). Among them, 15,013 (7.14%) were CLBC patients, and 195,396 (92.86%) were non-CLBC patients. From 2004 to 2015, the proportion of BCS was increased slightly in CLBC patients and relatively stable in non-CLBC patients (Fig. 2). The proportion of BCS in CLBC patients was lower than that of the non-CLBC group (42.7% vs. 60.5%, *p* < 0.001). The distribution of characteristics between CLBC and non-CLBC patients was significantly different except for chemotherapy (Supplementary Table 2).

The median age was 56 [interquartile range (IQR): 48 ~ 63 years]. The clinical characteristics of CLBC patients undergoing BCT and mastectomy are summarized in Table 1. Compared with patients who received a mastectomy, elderly patients, patients with a histological type of IDC, and the white race were more likely to receive BCS. In addition, CLBC patients with less aggressive characteristics such as lower histological grade, smaller tumor size, none or limited lymph node metastasis, or positive ER or PR status tended to be treated with BCS. CLBC patients treated with BCS were less likely to receive chemotherapy than mastectomy. For CLBC patients, the baseline clinicopathological characteristics of 4865 paired patients were balanced after the PSM (Table 1).

**Table 1:** Comparison of baseline features between BCS and mastectomy in CLBC patients before and after PSM [n (%)]

Characteristics	Before PSM			After PSM		
	Mastectomy *N* = 8597	BCS *N* = 6416	*P* value	Mastectomy *N* = 4865	BCS *N* = 4865	*P* value
Year of diagnosis						
2004–2007	2894 (33.7)	2178 (33.9)	0.008	1746 (35.9)	1758 (36.1)	0.826
2008–2011	2260 (26.3)	1551 (24.2)		1056 (21.7)	1031 (21.2)	
2012–2015	3443 (40.0)	2687 (41.9)		2063 (42.4)	2076 (42.7)	
Age at diagnosis, yrs						
18–40	794 (9.2)	275 (4.3)	< 0.001	153 (3.1)	159 (3.3)	0.941
41–50	2178 (25.3)	1234 (19.2)		906 (18.6)	913 (18.8)	
51–60	2812 (32.7)	2304 (35.9)		1733 (35.6)	1748 (35.9)	
61–70	2813 (32.7)	2603 (40.6)		2073 (42.6)	2045 (42.0)	
Race						
White	6609 (76.9)	5188 (80.9)	< 0.001	4260 (87.6)	4223 (86.8)	0.495
Black	805 (9.4)	626 (9.8)		258 (5.3)	293 (6.0)	
Other	1151 (13.4)	573 (8.9)		344 (7.1)	346 (7.1)	
Unknown	32 (0.4)	29 (0.5)		3 (0.1)	3 (0.1)	
Marital status						
Unmarried	3000 (34.9)	2159 (33.7)	0.03	1536 (31.6)	1529 (31.4)	0.877
Married	5253 (61.1)	4038 (62.9)		3259 (67.0)	3260 (67.0)	
Unknown	344 (4.0)	219 (3.4)		70 (1.4)	76 (1.6)	
Histological type						
IDC	6305 (73.3)	4827 (75.2)	< 0.001	3915 (80.5)	3898 (80.1)	0.903
ILC	785 (9.1)	434 (6.8)		250 (5.1)	252 (5.2)	
Others	1507 (17.5)	1155 (18.0)		700 (14.4)	715 (14.7)	
Grade						
G1	1407 (16.4)	1626 (25.3)	< 0.001	1291 (26.5)	1292 (26.6)	0.907
G2	3861 (44.9)	2949 (46.0)		2367 (48.7)	2353 (48.4)	
G3/4	2932 (34.1)	1555 (24.2)		1094 (22.5)	1096 (22.5)	
Unknown	397 (4.6)	286 (4.5)		113 (2.3)	124 (2.5)	
Tumor stage, AJCC 6^th^						
T1	4439 (51.6)	4927 (76.8)	< 0.001	3779 (77.7)	3807 (78.3)	0.509
T2	4158 (48.4)	1489 (23.2)		1086 (22.3)	1058 (21.7)	
Node stage, AJCC 6^th^						
N0	4667 (54.3)	4773 (74.4)	< 0.001	3707 (76.2)	3720 (76.5)	0.759
N1	2801 (32.6)	1411 (22.0)		1057 (21.7)	1031 (21.2)	
N2	777 (9.0)	172 (2.7)		80 (1.6)	89 (1.8)	
N3	352 (4.1)	60 (0.9)		21 (0.4)	25 (0.5)	
ER status						
Negative	1368 (15.9)	767 (12.0)	< 0.001	460 (9.5)	478 (9.8)	0.531
Positive	6943 (80.8)	5528 (86.2)		4356 (89.5)	4347 (89.4)	
Unknown	286 (3.3)	121 (1.9)		49 (1.0)	40 (0.8)	
PR status						
Negative	2319 (27.0)	1417 (22.1)	< 0.001	825 (17.0)	858 (17.6)	0.56
Positive	5915 (68.8)	4817 (75.1)		3979 (81.8)	3953 (81.3)	
Unknown	363 (4.2)	182 (2.8)		61 (1.3)	54 (1.1)	
HER2 status						
Negative	3189 (37.1)	2651 (41.3)	< 0.001	2082 (42.8)	2080 (42.8)	0.923
Positive	770 (9.0)	384 (6.0)		207 (4.3)	215 (4.4)	
Unknown	4638 (53.9)	3381 (52.7)		2576 (52.9)	2570 (52.8)	
Chemotherapy						
No/Unknown	3826 (44.5)	3782 (58.9)	< 0.001	3036 (62.4)	3028 (62.2)	0.884
Yes	4771 (55.5)	2634 (41.1)		1829 (37.6)	1837 (37.8)	

*Abbreviations: AJCC* American Joint Committee on Cancer, *BCS* Breast conserving surgery, *BCSS* Breast-cancer specific survival, *CLBC* Centrally located breast cancer, *ER* Estrogen receptor, *HER2* Human epidermal growth factor receptor-2, *IDC* Invasive ductal carcinoma, *ILC* Invasive lobular carcinoma, *OS* Overall survival, *PR* Progesterone receptor, *PSM* Propensity score matching, *CI* Confidence interval, *HR* Hazard ratio

The clinical characteristics of CLBC patients and non-CLBC cases treated with BCT were also compared (Table 2). Compared with non-CLBC patients, younger people with less aggressive characteristics such as lower histologic grade, smaller tumor size, none or limited lymph node metastasis, and positive ER or PR status, or negative HER-2 in CLBC cohorts were more likely to receive mastectomy, instead of BCS. However, the baseline clinicopathological characteristics of 6416 paired patients were balanced after the PSM (Table 2).

**Table 2:** Comparison of baseline features between CLBC and non-CLBC patients who received BCT before and after PSM

**Features**	Before PSM			After PSM		
	non-CLBC *N* = 118,211	CLC *N* = 6416	*P* value	non-CLBC *N* = 6416	CLBC *N* = 6416	*P* value
Year of diagnosis						
2004–2007	36,599 (31.0)	2178 (33.9)	< 0.001	2197 (34.2)	2178 (33.9)	0.899
2008–2011	28,133 (23.8)	1551 (24.2)		1531 (23.9)	1551 (24.2)	
2012–2015	53,479 (45.2)	2687 (41.9)		2688 (41.9)	2687 (41.9)	
Age at diagnosis, yrs						
18–40	6347 (5.4)	275 (4.3)	< 0.001	271 (4.2)	275 (4.3)	0.963
41–50	27,260 (23.1)	1234 (19.2)		1222 (19.0)	1234 (19.2)	
51–60	41,748 (35.3)	2304 (35.9)		2291 (35.7)	2304 (35.9)	
61–70	42,856 (36.3)	2603 (40.6)		2632 (41.0)	2603 (40.6)	
Race						
White	95,056 (80.4)	5188 (80.9)	0.316	5257 (81.9)	5188 (80.9)	0.159
Black	12,333 (10.4)	626 (9.8)		607 (9.5)	626 (9.8)	
Other	10,356 (8.8)	573 (8.9)		535 (8.3)	573 (8.9)	
Unknown	466 (0.4)	29 (0.5)		17 (0.3)	29 (0.5)	
Marital status						
Unmarried	39,066 (33.0)	2159 (33.7)	0.607	2162 (33.7)	2159 (33.7)	0.839
Married	75,063 (63.5)	4038 (62.9)		4047 (63.1)	4038 (62.9)	
Unknown	4082 (3.5)	219 (3.4)		207 (3.2)	219 (3.4)	
Histological type						
IDC	93,211 (78.9)	4827 (75.2)	< 0.001	4866 (75.8)	4827 (75.2)	0.427
ILC	7496 (6.3)	434 (6.8)		449 (7.0)	434 (6.8)	
Others	17,504 (14.8)	1155 (18.0)		1101 (17.2)	1155 (18.0)	
Grade						
G1	31,186 (26.4)	1626 (25.3)	< 0.001	1586 (24.7)	1626 (25.3)	0.576
G2	48,523 (41.0)	2949 (46.0)		2948 (45.9)	2949 (46.0)	
G3/4	34,729 (29.4)	1555 (24.2)		1612 (25.1)	1555 (24.2)	
Unknown	3773 (3.2)	286 (4.5)		270 (4.2)	286 (4.5)	
Tumor stage, AJCC 6^th^						
T1	89,048 (75.3)	4927 (76.8)	0.008	4892 (76.2)	4927 (76.8)	0.479
T2	29,163 (24.7)	1489 (23.2)		1524 (23.8)	1489 (23.2)	
Node stage, AJCC 6^th^						
N0	92,813 (78.5)	4773 (74.4)	< 0.001	4776 (74.4)	4773 (74.4)	0.218
N1	20,972 (17.7)	1411 (22.0)		1379 (21.5)	1411 (22.0)	
N2	3275 (2.8)	172 (2.7)		177 (2.8)	172 (2.7)	
N3	1151 (1.0)	60 (0.9)		84 (1.3)	60 (0.9)	
ER status						
Negative	19,487 (16.5)	767 (12.0)	< 0.001	753 (11.7)	767 (12.0)	0.665
Positive	97,003 (82.1)	5528 (86.2)		5554 (86.6)	5528 (86.2)	
Unknown	1721 (1.5)	121 (1.9)		109 (1.7)	121 (1.9)	
PR status						
Negative	30,499 (25.8)	1417 (22.1)	< 0.001	1427 (22.2)	1417 (22.1)	0.448
Positive	85,134 (72.0)	4817 (75.1)		4830 (75.3)	4817 (75.1)	
Unknown	2578 (2.2)	182 (2.8)		159 (2.5)	182 (2.8)	
HER2 status						
Negative	53,185 (45.0)	2651 (41.3)	< 0.001	2661 (41.5)	2651 (41.3)	0.927
Positive	7313 (6.2)	384 (6.0)		374 (5.8)	384 (6.0)	
Unknown	57,713 (48.8)	3381 (52.7)		3381 (52.7)	3381 (52.7)	
Chemotherapy						
No/Unknown	67,251 (56.9)	3782 (58.9)	0.001	3739 (58.3)	3782 (58.9)	0.452
Yes	50,960 (43.1)	2634 (41.1)		2677 (41.7)	2634 (41.1)	

*Abbreviations: AJCC* American Joint Committee on Cancer, *BCS* Breast conserving surgery, *ER* Estrogen receptor, *HER2* Human epidermal growth factor receptor-2, *IDC* Invasive ductal carcinoma, *ILC* Invasive lobular carcinoma, *PR* Progesterone receptor, *PSM* Propensity score matching

### Survival analyses before PSM

**Fig. 3:**
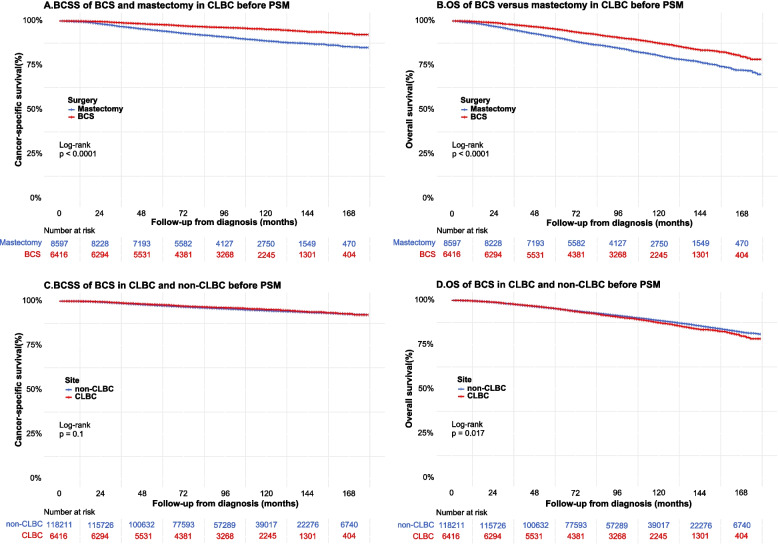
Survival curves of BCSS and OS stratified by surgery procedure and tumor location before PSM (**A** BCSS of BCS versus mastectomy; **B** OS of BCS versus mastectomy; **C** BCSS of CLBC versus non-CLBC; **D** OS of CLBC versus non-CLBC)

Overall, 15,013 CLBC cases and 195,396 non-CLBC cases were enrolled in the analysis (Supplementary Table 2). During a median follow-up of 91 months (IQR: 58 ~ 130 months), 24,687 patients died (11.73%) and 12,833 (6.10%) patients died of breast cancer in the total population. The Kaplan–Meier survival curves showed that CLBC patients who received BCS had a superior BCSS and OS than those patients who were treated with mastectomy (Fig. 3A, B). Meanwhile, there was no difference in BCSS and OS between CLBC and non-CLBC groups in patients treated with BCT (Fig. 3C, D). The estimated 3-, 5-, 7-, and 10-year BCSS rates and OS rates were summarized in Supplementary Table 3.

Among CLBC patients, the univariate Cox analysis found that year of diagnosis, age at diagnosis, race, marital status, grade, tumor stage, node stage, ER status, PR status, surgery procedure, and chemotherapy were associated with the BCSS and OS, while HER-2 status and radiotherapy were only associated with OS but not BCSS (Supplementary Table 4). After adjusting the potential confounding factors, the multivariable Cox regression model showed that patients who received BCS had a better BCSS (HR = 0.67, 95%CI: 0.55–0.80, *p* < 0.001) and OS (HR = 0.78, 95%CI:0.68–0.89, *p* = 0.0005) compared with patients who received mastectomy (Supplementary Table 5).

Among patients who received BCS, the univariate Cox analysis showed that the tumor location was not associated with BCSS but with OS (Supplementary Table 6). The multivariable Cox model showed that patients with CLBC had a similar BCSS (HR = 0.99, 95%CI: 0.87–1.12, *p* = 0.850) and a worse OS (HR = 1.09, 95%CI: 1.01–1.18, *p* = 0.030) compared with the non-CLBC patients (Supplementary Table 7).

### Survival analyses after PSM

**Fig. 4:**
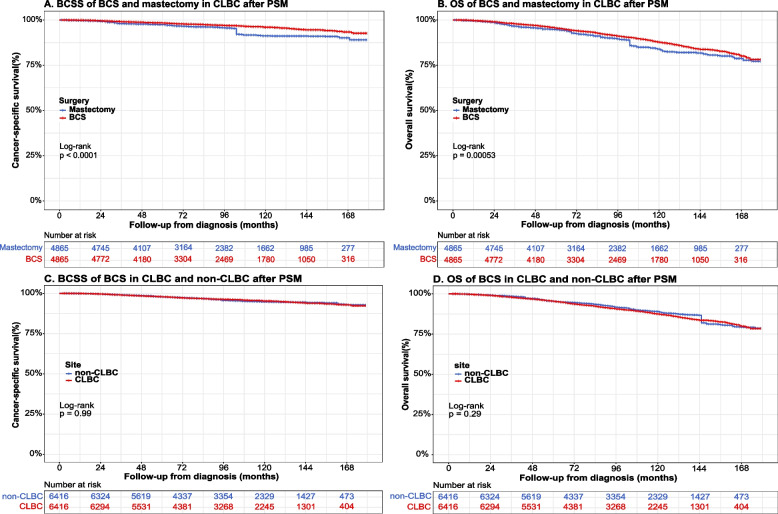
Survival curves of BCSS and OS stratified by surgery procedure and tumor location after PSM (**A** BCSS of BCS versus mastectomy; **B** OS of BCS versus mastectomy; **C** BCSS of CLBC versus non-CLBC; **D** OS of CLBC versus non-CLBC)

The survival curves showed that CLBC patients treated with BCS had a higher BCSS rate (log-rank test: *p* = 0.0005) and OS rate (log-rank test:* p* < 0.0001) compared to those who received mastectomy (Fig. 4A, B). Multivariable Cox model analyses showed that BCT was associated with a better BCSS (HR = 0.65, 95%CI: 0.47–0.90, *p* = 0.009) and OS (HR = 0.82, 95%CI: 0.65–1.04, *p* = 0.110) (Table 3) in patients with a CLBC.

**Table 3:** Multivariable Cox analysis for BCSS and OS in centrally located breast cancer patients after PSM

**Characteristic**	**No. at risk**	**BCSS**		**OS**	
		**HR (95%CI)**	***P* value**	**HR (95%CI)**	***P* value**
Surgery					
Mastectomy	4865	Reference		Reference	
BCS	4865	0.65(0.47–0.90)	**0.009**	0.82(0.65–1.04)	**0.109**
Year of diagnosis					
2004–2007	3504	Reference		Reference	
2008–2011	2087	0.51(0.38–0.68)	< 0.001	7.46(0.63–0.88)	0.0005
2012–2015	4139	0.36(0.21–0.62)	0.0002	0.42(0.31–0.59)	< 0.001
Age at diagnosis, yrs					
18–40	312	Reference		Reference	
41–50	1819	0.63(0.41–0.95)	0.026	0.87(0.60–1.26)	0.468
51–60	3481	0.63(0.43–0.94)	0.022	1.07(0.75–1.51)	0.717
61–70	4118	0.98(0.67–1.44)	0.937	2.22(1.57–3.12)	< 0.001
Race					
White	8483	Reference		Reference	
Black	551	1.68(1.21–2.33)	0.002	1.82(1.48–2.23)	< 0.001
Other	690	0.42(0.24–0.73)	0.002	7.82(0.59–1.03)	0.085
Unknown	6	NA	NA	NA	NA
Marital status					
Unmarried	3065	Reference		Reference	
Married	6519	1.28(1.04–1.58)	0.019	0.89(0.79–1.01)	0.079
Unknown	146	2.73(1.38–5.40)	0.004	1.69(1.06–2.68)	0.026
Histological type					
IDC	7813	Reference		Reference	
ILC	502	0.54(0.30–0.97)	0.038	1.03(0.77–1.37)	0.856
Others	1415	0.46(0.31–0.68)	0.0001	1.01(0.85–1.20)	0.916
Grade					
G1	2583	Reference		Reference	
G2	4720	3.80(2.51–5.74)	< 0.001	1.16(0.99–1.36)	0.065
G3/4	2190	6.82(4.41–10.55)	< 0.001	1.89(1.56–2.30)	< 0.001
Unknown	237	2.70(1.16–6.30)	0.022	0.98(0.65–1.47)	0.907
Tumor stage, AJCC 6th					
T1	7586	Reference		Reference	
T2	2144	2.12(1.72–2.61)	< 0.001	1.65(1.43–1.90)	< 0.001
Node stage, AJCC 6th					
N0	7427	Reference		Reference	
N1	2088	1.90(1.51–2.40)	< 0.001	1.97(1.54–2.10)	< 0.001
N2	169	4.50(3.05–6.66)	< 0.001	3.04(2.23–4.15)	< 0.001
N3	46	4.89(2.77–8.62)	< 0.001	3.21(1.95–5.28)	< 0.001
ER status					
Negative	938	Reference		Reference	
Positive	8703	1.00(0.71–1.43)	0.975	0.80(0.62–1.02)	0.070
Unknown	89	1.95(0.37–10.34)	0.434	0.88(0.33–2.34)	0.791
PR status					
Negative	1683	Reference		Reference	
Positive	7932	0.84(0.63–1.12)	0.242	0.92(0.75–1.12)	0.408
Unknown	115	0.42(0.08–2.17)	0.297	0.84(0.35–2.02)	0.701
HER2 status					
Negative	4162	Reference		Reference	
Positive	422	0.74(0.39–1.40)	0.360	0.94(0.61–1.44)	0.769
Unknown	5146	0.55(0.34–0.90)	0.016	0.68(0.52–0.90)	0.007
Radiotherapy					
No/Unknown	4275	Reference		Reference	
Yes	5455	0.80(0.57–1.10)	0.169	0.96(0.75–1.23)	0.770
Chemotherapy					
No/Unknown	6064	Reference		Reference	
Yes	3666	0.71(0.56–0.91)	0.006	0.64(0.54–0.75)	< 0.001

*Abbreviations: AJCC* American Joint Committee on Cancer, *BCS* Breast conserving surgery, *BCSS* Breast-cancer specific survival, *CI* Confidence interval, *ER* Estrogen receptor, *HER2* Human epidermal growth factor receptor-2, *HR* Hazard ratio, *IDC* Invasive ductal carcinoma, *ILC* Invasive lobular carcinoma, *OS* Overall survival, *PR* Progesterone receptor, *PSM* Propensity score matching

The survival curves showed that the BCSS (log-rank test: *p* = 0.99) and OS (log-rank test: *p* = 0.29) were similar between CLBC and non-CLBC patients who received BCS (Fig. 4C, D). The multivariable Cox model showed that patients with CLBC who received BCS had a similar BCSS (HR = 1.05, 95%CI: 0.88–1.24, *p* = 0.614) and OS (HR = 1.08, 95%CI: 0.97–1.20, *p* = 0.168) compared to patients with non-CLBC treated with BCS (Table 4).

**Table 4:** Multivariable Cox analysis for BCSS and OS in patients who received breast-conserving treatment after PSM

**Characteristics**	**BCSS**		**OS**	
	**HR (95%CI)**	***P* value**	**HR (95%CI)**	***P* value**
Year of diagnosis				
2004–2007	Reference		Reference	
2008–2011	0.98(0.78–1.25)	0.896	1.19(1.03–1.38)	0.019
2011–2015	0.93(0.62–1.41)	0.740	1.38(1.06–1.80)	0.017
Age at diagnosis, yrs				
18–40	Reference		Reference	
41–50	0.82(0.59–1.16)	0.267	0.88(0.65–1.18)	0.388
51–60	0.72(0.52–1.00)	0.050	1.05(0.79–1.38)	0.745
61–70	0.80(0.58–1.12)	0.198	1.98(1.51–2.61)	< 0.001
Race				
White	Reference		Reference	
Black	1.56(1.24–1.97)	< 0.001	1.52(1.30–1.78)	< 0.001
Other	0.91(0.65–1.29)	0.607	0.89(0.72–1.11)	0.306
Unknown	0.84(0.12–5.99)	0.861	0.30(0.04–2.10)	0.223
Marital status				
Unmarried	Reference		Reference	
Married	0.75(0.63–0.90)	0.002	0.77(0.69–0.86)	< 0.001
Unknown	0.73(0.42–1.28)	0.268	0.68(0.48–0.97)	0.035
Histological type				
IDC	Reference		Reference	
ILC	1.06(0.73–1.55)	0.748	0.85(0.67–1.08)	0.189
Others	0.86(0.66–1.11)	0.245	0.80(0.69–0.93)	0.004
Grade				
G1	Reference		Reference	
G2	2.16(1.5–3.09)	< 0.001	0.97(0.84–1.12)	0.672
G3/4	3.65(2.51–5.3)	< 0.001	1.35(1.14–1.60)	< 0.001
Unknown	2.86(1.66–4.92)	< 0.001	1.04(0.77–1.40)	0.785
Tumor stage, AJCC 6^th^				
T1	Reference		Reference	
T2	2.20(1.82–2.66)	< 0.001	1.68(1.48–1.90)	< 0.001
Node stage, AJCC 6^th^				
N0	Reference		Reference	
N1	2.00(1.62–2.47)	< 0.001	1.56(1.36–1.79)	< 0.001
N2	3.04(2.19–4.23)	< 0.001	2.32(1.81–2.98)	< 0.001
N3	5.96(4.14–8.58)	< 0.001	3.85(2.83–5.23)	< 0.001
ER status				
Negative	Reference		Reference	
Positive	1.02(0.76–1.37)	0.882	0/76(0.62–0.94)	0.009
Unknown	1.06(0.36–3.17)	0.911	0.63(0.34–1.17)	0.145
PR status				
Negative	Reference		Reference	
Positive	0.66(0.52–0.85)	0.001	1.00(0.85–1.19)	0.968
Unknown	0.74(0.28–1.93)	0.538	1.18(0.72–1.93)	0.513
HER2 status				
Negative	Reference		Reference	
Positive	0.72(0.46–1.12)	0.146	0.89(0.64–1.23)	0.470
Unknown	0.86(0.60–1.23)	0.412	1.12(0.89–1.41)	0.349
Chemotherapy				
No/Unknown	Reference		Reference	
Yes	1.20(0.95–1.52)	0.126	0.76(0.67–0.88)	< 0.001
Tumor location				
Non-central	Reference		Reference	
Central	1.05(0.88–1.24)	0.615	1.08(0.97–1.20)	0.168

*Abbreviations: AJCC* American Joint Committee on Cancer, *BCS* Breast conserving surgery, *BCSS* Breast-cancer specific survival, *CI* Confidence interval, *ER* Estrogen receptor, *HER2* Human epidermal growth factor receptor-2, *HR* Hazard ratio, *IDC* Invasive ductal carcinoma, *ILC* Invasive lobular carcinoma, *OS* Overall survival, *PR* Progesterone receptor, *PSM* Propensity score matching

## Discussion

The choice between mastectomy and BCS refers to the research evidence, cosmetic appearance, and patient’s desire. In our study, only 42.7% of CLBC patients underwent BCS, which is significantly less than that of non-CLBC patients (60.5%). However, the survival analysis results showed that there was no difference in OS and BCSS between BCS and mastectomy in the CLBC populations, and between CLBC patients and non-CLBC patients who received BCS. Therefore, there is rising confidence in advocating BCS as a surgical option for women with early-stage CLBC from a safety perspective.

Some differences in clinicopathological features were observed between CLBC and non-CLBC patients. In the CLBC population, these patients who exhibited less aggressive histological traits tended to receive BCS. This may be one reason for the therapeutic difference between the two populations. Unfortunately, we were unable to access the detailed information on adjuvant therapy or other pathological features (Ki-67 et al.) in the SEER database, which limited us to determine whether these factors would influence the decision of breast surgery [22, 23]. Compared with non-CLBC, patients with less aggressive characteristics such as lower histologic grade, smaller tumor size, none or limited lymph node metastasis, positive hormone receptor status, or negative HER-2 in CLBC cohorts were more likely to receive mastectomy, instead of BCS. The tumor characteristics of CLBC patients who received BCS are not completely superior to those of non-CLBC patients. Interestingly, our survival data supported that BCS for CLBC resulted in non-inferior outcomes compared with mastectomy in CLBC or with BCS in non-CLBC, even in N2-N3 disease. These indicated that tumor biological behavior features should not be a barrier to BCS in CLBC. The improved survival for BCS was likely attributed to the advances in early diagnosis, surgery procedures, and adjuvant therapy over the last decades [24, 25].

The standard implementation of BCS and adjuvant therapy is still the basic guarantee of BCT for patients with CLBC. There is no special requirement for a negative margin for BCS in CLBC patients. However, we need to be aware that central breast cancer may have the potential to invade the wider breast ducts [8, 9]. Considering the cosmetic appearance and local control, tumor size is an important factor associated with the selection of BCS in clinical practice, which is supported by several guidelines and consensuses [1, 2, 26]. Several small sample, short-term follow-up studies based on the SEER database have enrolled some T3-4 or stage III/IV disease which would influence the application of research results [14, 15, 17, 18]. Adjuvant radiotherapy was necessary for reducing local recurrence and improving long-term disease-free survival and overall survival for whole breast cancer populations treated with BCS [27–29]. Without radiotherapy, the nipple-areola complex involvement was related to a higher risk of local recurrence after BCS [30]. In previous reported studies, many patients treated with BCS but without radiotherapy, which would induce a select bias [15–18]. Based on the consideration of clinical practice, the tumor stage was limited as T1-2 and radiotherapy after BCS was required in our study, which makes the study results suitable for clinical work.

CLBC has a four times higher risk of involvement of nipple-areolar complex (NAC) than that of non-CLBC [31]. Removal of NAC is usually recommended for BCS in CLBC patients. However, traditional incisions with NAC resection usually led to poor aesthetic outcomes [32, 33]. Preserving NAC is generally considered essential for maintaining the aesthetic appearance of the breast. Oncoplastic techniques for breast-conserving surgery could improve cosmetic results without jeopardizing oncological outcomes [34]. A good cosmetic outcome improves psychosocial adjustment after breast cancer treatment. Fortunately, many articles have discussed various approaches to achieve optimal results following oncoplastic surgery, ranging from classic reduction mammaplasty to different flap reconstruction techniques [35–37]. Additionally, restoration of the soft tissue defects by various oncoplastic procedures, with or without immediate reconstruction of the NAC, has been proven to be both oncological safe and cosmetically effective [10, 35]. So, the aesthetic factor should not be the main obstacle to the performance of BCS in CLBC patients.

In this study, many clinicopathological features were identified as independent prognostic factors for BCSS and OS. Patients with older age, married, lower grade, smaller tumor size, lymph node-negative, hormone receptor-positive, HER-2 negative, and BCS were the greater independent prognostic factor of BCSS and OS for CLBC. Meanwhile, chemotherapy was a superior prognostic indicator for OS. Contrastingly, radiotherapy did not improve survival in the whole CLBC population. Similar results were observed in CLBC patients who received BCS. Tumor stage (tumor size, lymph node stage), biological features (grade, hormone receptor status, HER2 status), and chemotherapy have been investigated well to predict survival for breast cancer patients [24, 25, 38, 39]. Marital status reflects the social, psychological, and economic conditions, which could affect the diagnosis, treatment, and prognosis of a female patient indirectly [40, 41]. The results of these prognostic analyses were well consistent with those of previous reports, which indicated the results of our study have high reliability. Identifying these risk factors is instrumental to accurately assess prognosis and develop individualized management strategies [42, 43].

There are still some limitations in our research. First, although we have set inclusion and exclusion criteria strictly, and taken the PSM method to balance the baseline features, there may still exist some selective bias. Second, local recurrence data are unavailable from the SEER database, so we cannot evaluate the recurrence between each group. Third, we could not evaluate the influence of neoadjuvant chemotherapy on surgical choice and survival outcomes. Fourth, we did not have access to detailed information on adjuvant therapy, which may influence the survival outcomes. Fifth, the lack of information on comorbidities, performance status, treatment toxicities, tumor biological features (Ki-67 rate), education level, and socioeconomic status which may introduce bias into our results. In addition, we could not obtain data about the cosmetic results and satisfaction with body image after BCT.

## Conclusion

Our study demonstrated the long-term oncological safety of BCS in CLBC compared with mastectomy and non-CLBC disease. As a result, BCS should be an acceptable and preferable alternative to mastectomy for well-selected, early-stage CLBC patients.

### Supplementary Information

**Additional file 1: Supplemental Table 1.** The surgical procedure and corresponding the site-specific surgery codes. **Supplemental Table 2.** Comparison of baseline features between central and non-central breast cancer patients. **Supplemental Table 3.** The estimated 3-, 5-, 7-, and 10-year BCSS rate and OS rate in central and outer breast cancer patients with different surgical procedure. **Supplemental Table 4.** Univariate cox analysis of prognosis of breast cancer in central region before PSM. **Supplemental Table 5.** Multivariable Cox analysis for BCSS and OS in centrally located breast cancer patients before PSM. **Supplemental Table 6.** Univariate cox analysis of BCSS and OS in breast-conserving patients with central and non-central tumor before PSM. **Supplemental Table 7.** Multivariable Cox analysis for BCSS and OS in patients received breast-conserving treatment before PSM.

## Data Availability

The datasets generated and analyzed in this study are available in the SEER repository (https://seer.cancer.gov/). If need more detailed data, you can contact the corresponding author further.
