# Supplementary material for: Breast-conserving surgery is an appropriate procedure for centrally located breast cancer: a population-based retrospective cohort study
Source: BMC Surg. 2023 Oct 3;23:298. doi: 10.1186/s12893-023-02181-6 (PMC10548734; doi:10.1186/s12893-023-02181-6)
Supplement: Supplementary file 1 — Additional file 1: Supplemental Table 1. The surgical procedure and corresponding the site-specific surgery codes. Supplemental Table 2. Comparison of baseline features between central and non-central breast cancer patients. Supplemental Table 3. The estimated 3-, 5-, 7-, and 10-year BCSS rate and OS rate in central and outer breast cancer patients with different surgical procedure. Supplemental Table 4. Univariate cox analysis of prognosis of breast cancer in central region before PSM. Supplemental Table 5. Multivariable Cox analysis for BCSS and OS in centrally located breast cancer patients before PSM. Supplemental Table 6. Univariate cox analysis of BCSS and OS in breast-conserving patients with central and non-central tumor before PSM. Supplemental Table 7. Multivariable Cox analysis for BCSS and OS in patients received breast-conserving treatment before PSM. [file 12893_2023_2181_MOESM1_ESM.docx]

**Supplement materials**

**Contents:**

**Supplemental table 1. The surgical procedure and corresponding the site-specific surgery codes**

**Supplemental table 2. Comparison of baseline features between central and non-central breast cancer patients**

**Supplemental table 3. The estimated 3-, 5-, 7-, and 10-year BCSS rate and OS rate in central and outer breast cancer patients with different surgical procedure.**

**Supplemental table 4. Univariate cox analysis of prognosis of breast cancer in central region before PSM**

**Supplemental table 5. Multivariable Cox analysis for BCSS and OS in centrally located breast cancer patients before PSM**

**Supplemental table 6. Univariate cox analysis of BCSS and OS in breast-conserving patients with central and non-central tumor before PSM.**

**Supplemental table 7. Multivariable Cox analysis for BCSS and OS in patients received breast-conserving treatment before PSM.**

**Supplemental table 1. The surgical procedure and corresponding the site-specific surgery codes**

| **Surgical procedure** | **The site-specific surgery codes in SEER database** |
| --- | --- |
| **BCS** | Code 19  Code 20 |
| **Mastectomy** | Code from 30 to 76  Code 80 |

**Abbreviation:** BCS: breast-conserving surgery.

**Supplemental table 2. Comparison of baseline features between central and non-central breast cancer patients**

| Characteristics | Outer N=195396 | Center N=15013 | P value |
| --- | --- | --- | --- |
| Year of diagnosis |  |  |  |
| 2004-2007 | 58431 (29.9) | 5072 (33.8) | <0.001 |
| 2008-2011 | 47735 (24.4) | 3811 (25.4) |  |
| 2012-2015 | 89230 (45.7) | 6130 (40.8) |  |
| Age at diagnosis, yrs |  |  |  |
| 18-40 | 16000 (8.2) | 1069 (7.1) | <0.001 |
| 41-50 | 50322 (25.8) | 3412 (22.7) |  |
| 51-60 | 65831 (33.7) | 5116 (34.1) |  |
| 61-70 | 63243 (32.4) | 5416 (36.1) |  |
| Race |  |  |  |
| White | 154720 (79.2) | 11797 (78.6) | <0.001 |
| Black | 20920 (10.7) | 1431 (9.5) |  |
| Other | 18951 (9.7) | 1724 (11.5) |  |
| Unknown | 805 (0.4) | 61 (0.4) |  |
| Marital status |  |  |  |
| Unmarried | 64801 (33.2) | 5159 (34.4) | 0.005 |
| Married | 123521 (63.2) | 9291 (61.9) |  |
| Unknown | 7074 (3.6) | 563 (3.8) |  |
| Histological type |  |  |  |
| IDC | 152121 (77.9) | 11132 (74.1) | <0.001 |
| ILC | 13938 (7.1) | 1219 (8.1) |  |
| Others | 29337 (15.0) | 2662 (17.7) |  |
| Grade |  |  |  |
| G1 | 44085 (22.6) | 3033 (20.2) | <0.001 |
| G2 | 79628 (40.8) | 6810 (45.4) |  |
| G3/4 | 64872 (33.2) | 4487 (29.9) |  |
| Unknown | 6811 (3.5) | 683 (4.5) |  |
| Tumor stage, AJCC 6th |  |  |  |
| T1 | 131454 (67.3) | 9366 (62.4) | <0.001 |
| T2 | 63942 (32.7) | 5647 (37.6) |  |
| Node stage, AJCC 6th |  |  |  |
| N0 | 139127 (71.2) | 9440 (62.9) | <0.001 |
| N1 | 43100 (22.1) | 4212 (28.1) |  |
| N2 | 9334 (4.8) | 949 (6.3) |  |
| N3 | 3835 (2.0) | 412 (2.7) |  |
| ER status |  |  |  |
| Negative | 36502 (18.7) | 2135 (14.2) | <0.001 |
| Positive | 154973 (79.3) | 12471 (83.1) |  |
| Unknown | 3921 (2.0) | 407 (2.7) |  |
| PR status |  |  |  |
| Negative | 55015 (28.2) | 3736 (24.9) | <0.001 |
| Positive | 135045 (69.1) | 10732 (71.5) |  |
| Unknown | 5336 (2.7) | 545 (3.6) |  |
| HER2 status |  |  |  |
| Negative | 85775 (43.9) | 5840 (38.9) | <0.001 |
| Positive | 14932 (7.6) | 1154 (7.7) |  |
| Unknown | 94689 (48.5) | 8019 (53.4) |  |
| Surgery |  |  |  |
| Mastectomy | 77185 (39.5) | 8597 (57.3) | <0.001 |
| BCS | 118211 (60.5) | 6416 (42.7) |  |
| Radiotherapy |  |  |  |
| No/Unknown | 58393 (29.9) | 6530 (43.5) | <0.001 |
| Yes | 137003 (70.1) | 8483 (56.5) |  |
| Chemotherapy |  |  |  |
| No/Unknown | 100569 (51.5) | 7608 (50.7) | 0.062 |
| Yes | 94827 (48.5) | 7405 (49.3) |  |

**Abbreviation:** AJCC, American Joint Committee on Cancer; BCS, breast conserving surgery; ER, estrogen receptor; HER2, human epidermal growth factor receptor-2; IDC, invasive ductal carcinoma; ILC, invasive lobular carcinoma; PR, progesterone receptor.

**Supplemental table 3**. The estimated 3-, 5-, 7-, and 10-year BCSS rate and OS rate in central and outer breast cancer patients with different surgical procedure.

| Cohorts | NO. at risk | Estimated survival rate (95%*CI*), % | | | |
| --- | --- | --- | --- | --- | --- |
|  |  | 3-year | 5-year | 7-year | 10-year |
| **BCSS** |  |  |  |  |  |
| BCS in central | 6416 | 98.9(98.7-99.2) | 97.9(97.5-98.3) | 96.7(96.2-97.2) | 95.2(94.6-95.9) |
| Mastectomy in central | 8597 | 96.8(96.4-97.2) | 94.3(93.8-94.8) | 92.0(91.3-92.6) | 88.7(87.9-89.6) |
| BCS in non-central | 118211 | 98.7(98.7-98.8) | 97.4(97.3-97.5) | 96.2(96.1-94.8) | 94.6(94.5-94.8) |
| **OS** |  |  |  |  |  |
| BCS in central | 6416 | 97.8(97.4-98.2) | 95.36(94.8-95.9) | 92.3(91.6-93.0) | 87.42(86.4-88.4) |
| Mastectomy in central | 8597 | 94.8(94.4-95.3) | 90.6(90.0-91.3) | 86.5(85.7-87.3) | 80.4(79.4-81.4) |
| BCS in non-central | 118211 | 97.7(97.6-97.8) | 95.3(95.2-95.4) | 92.8(92.6-92.9) | 88.6(88.4-88.9) |

**Abbreviation:** BCS, breast conserving surgery; BCSS, breast-cancer specific survival; CI, confidence interval; OS, overall survival.

**Supplemental table 4. Univariate cox analysis of prognosis of breast cancer in central region before PSM**

| **Characteristic** | **BCSS** | |  | **OS** | |
| --- | --- | --- | --- | --- | --- |
|  | **HR (95%CI)** | **P value** |  | **HR (95%CI)** | **P value** |
| Year of diagnosis |  |  |  |  |  |
| 2004-2007 | Reference |  |  | Reference |  |
| 2008-2011 | 0.97(0.84-1.13) | 0.711 |  | 097(0.87-1.07) | 0.526 |
| 2012-2015 | 0.73(0.61-0.86) | 0.0002 |  | 0.82(0.72-0.92) | 0.001 |
| Age at diagnosis, yrs |  |  |  |  |  |
| 18-40 | Reference |  |  | Reference |  |
| 41-50 | 0.56(0.44-0.69) | <0.001 |  | 0.72(0.59-0.88) | 0.001 |
| 51-60 | 0.60(0.49-0.74) | <0.001 |  | 0.96(0.80-1.15) | 0.664 |
| 61-70 | 0.61(0.50-0.75) | <0.001 |  | 1.64(1.38-1.96) | <0.001 |
| Race |  |  |  |  |  |
| White | Reference |  |  | Reference |  |
| Black | 1.74(1.47-2.07) | <0.001 |  | 1.60(1.42-1.81) | <0.001 |
| Other | 0.93(0.75-1.14) | 0.459 |  | 0.78(0.67-0.91) | 0.001 |
| Unknown | 0.25(0.03-1.75) | 0.161 |  | 0.12(0.02-0.82) | 0.031 |
| Marital status |  |  |  |  |  |
| Unmarried | Reference |  |  | Reference |  |
| Married | 0.72(0.63-0.81) | <0.001 |  | 0.62(0.57-0.68) | <0.001 |
| Unknown | 0.78(0.55-1.11) | 0.161 |  | 0.70(0.55-0.90) | 0.006 |
| Histological type |  |  |  |  |  |
| IDC | Reference |  |  | Reference |  |
| ILC | 0.98(0.79-1.23) | 0.867 |  | 1.23(0.97-1.31) | 0.118 |
| Others | 0.70(0.58-0.83) | <0.001 |  | 0.88(0.78-0.99) | 0.028 |
| Grade |  |  |  |  |  |
| G1 | Reference |  |  | Reference |  |
| G2 | 2.61(2.02-3.39) | <0.001 |  | 1.26(1.10-1.43) | <0.001 |
| G3/4 | 5.91(4.58-7.61) | <0.001 |  | 2.01(1.77-2.29) | <0.001 |
| Unknown | 2.17(1.44-3.27) | 0.0002 |  | 1.17(0.92-1.48) | 0.192 |
| Tumor stage, AJCC 6th |  |  |  |  |  |
| T1 | Reference |  |  | Reference |  |
| T2 | 3.74(3.29-4.26) | <0.001 |  | 2.15(1.98-2.34) | <0.001 |
| Node stage, AJCC 6th |  |  |  |  |  |
| N0 | Reference |  |  | Reference |  |
| N1 | 2.47(2.14-2.86) | <0.001 |  | 1.63(1.48-1.79) | <0.001 |
| N2 | 5.59(4.68-6.68) | <0.001 |  | 3.02(2.65-3.45) | <0.001 |
| N3 | 10.38(8.49-12.69) | <0.001 |  | 4.82(4.09-5.67) | <0.001 |
| ER status |  |  |  |  |  |
| Negative | Reference |  |  | Reference |  |
| Positive | 0.51(0.44-0.59) | <0.001 |  | 0.65(0.59-0.73) | <0.001 |
| Unknown | 0.69(0.49-0.96) | 0.027 |  | 0.73(0.57-0.93) | 0.012 |
| PR status |  |  |  |  |  |
| Negative | Reference |  |  | Reference |  |
| Positive | 0.49(0.43-0.56) | <0.001 |  | 0.66(0.60-0.72) | <0.001 |
| Unknown | 0.67(0.50-0.90) | 0.008 |  | 0.75(0.61-0.92) | 0.007 |
| HER2 status |  |  |  |  |  |
| Negative | Reference |  |  | Reference |  |
| Positive | 1.23(0.93-1.64) | 0.149 |  | 1.09(0.88-1.36) | 0.415 |
| Unknown | 1.16(0.99-1.35) | 0.061 |  | 1.12(1.00-1.25) | 0.046 |
| Surgery |  |  |  |  |  |
| Mastectomy | Reference |  |  | Reference |  |
| BCS | 0.42(0.36-0.48) | <0.001 |  | 0.61(0.56-0.67) | <0.001 |
| Radiotherapy |  |  |  |  |  |
| No/Unknown | Reference |  |  | Reference |  |
| Yes | 0.97(0.86-1.10) | 0.658 |  | 0.88(0.81-0.96) | 0.003 |
| Chemotherapy |  |  |  |  |  |
| No/Unknown | Reference |  |  | Reference |  |
| Yes | 2.49(2.18-2.84) | <0.001 |  | 1.25(1.15-1.36) | <0.001 |

**Abbreviation:** AJCC, American Joint Committee on Cancer; BCS, breast conserving surgery; BCSS, breast-cancer specific survival; CI, confidence interval; ER, estrogen receptor; HER2, human epidermal growth factor receptor-2; HR, hazard ratio; IDC, invasive ductal carcinoma; ILC, invasive lobular carcinoma; OS, overall survival; PR, progesterone receptor; PSM, propensity score matching.

**Supplemental table 5. Multivariable Cox analysis for BCSS and OS in centrally located breast cancer patients before PSM**

| Characteristic | BCSS | |  | OS | |
| --- | --- | --- | --- | --- | --- |
|  | *HR* (95%*CI*) | *P* value |  | *HR* (95%*CI*) | *P* value |
| Year of diagnosis |  |  |  |  |  |
| 2004-2007 | Reference |  |  | Reference |  |
| 2008-2011 | 0.91(0.77-1.08) | 0.274 |  | 0.94(0.84-1.06) | 0.321 |
| 2012-2015 | 0.58(0.44-0.77) | 0.0001 |  | 0.75(0.61-0.92) | 0.005 |
| Age at diagnosis, yrs |  |  |  |  |  |
| 18-40 | Reference |  |  | Reference |  |
| 41-50 | 0.72(0.58-0.90) | 0.004 |  | 0.84(0.69-1.03) | 0.091 |
| 51-60 | 0.84(0.68-1.04) | 0.102 |  | 1.15(0.96-1.39) | 0.133 |
| 61-70 | 0.96(0.78-1.18) | 0.694 |  | 2.06(1.72-2.46) | <0.001 |
| Race |  |  |  |  |  |
| White | Reference |  |  | Reference |  |
| Black | 1.40(1.18-1.67) | 0.0001 |  | 1.43(1.26-1.62) | <0.001 |
| Other | 0.81(0.66-1.00) | 0.052 |  | 0.78(0.67-0.91) | 0.001 |
| Unknown | 0.33(0.046-2.32) | 0.262 |  | 0.14(0.02-1.01) | 0.052 |
| Marital status |  |  |  |  |  |
| Unmarried | Reference |  |  | Reference |  |
| Married | 0.80(0.70-0.91) | 0.0006 |  | 0.72(0.66-0.78) | <0.001 |
| Unknown | 0.87(0.61-1.23) | 0.429 |  | 0.78(0.61-1.00) | 0.054 |
| Histological type |  |  |  |  |  |
| IDC | Reference |  |  | Reference |  |
| ILC | 1.13(0.90-1.43) | 0.299 |  | 1.13(0.97-1.32) | 0.117 |
| Others | 0.83(0.69-0.99) | 0.040 |  | 0.93(0.83-1.05) | 0.244 |
| Grade |  |  |  |  |  |
| G1 | Reference |  |  | Reference |  |
| G2 | 1.79(1.37-2.32) | <0.001 |  | 1.07(0.93-1.22) | 0.344 |
| G3/4 | 2.86(2.19-3.75) | <0.001 |  | 1.44(1.25-1.67) | <0.001 |
| Unknown | 1.69(1.12-2.55) | 0.013 |  | 1.06(0.84-1.35) | 0.608 |
| Tumor stage, AJCC 6th |  |  |  |  |  |
| T1 | Reference |  |  | Reference |  |
| T2 | 2.05(1.78-2.36) | <0.001 |  | 1.63(1.48-1.79) | <0.001 |
| Node stage, AJCC 6th |  |  |  |  |  |
| N0 | Reference |  |  | Reference |  |
| N1 | 1.87(1.60-2.19) | <0.001 |  | 1.60(1.44-1.78) | <0001 |
| N2 | 3.35(2.71-4.12) | <0.001 |  | 2.70(2.31-3.16) | <0.001 |
| N3 | 5.46(4.35-6.84) | <0.001 |  | 3.85(3.21-4.62) | <0.001 |
| ER status |  |  |  |  |  |
| Negative | Reference |  |  | Reference |  |
| Positive | 0.95(0.79-1.16) | 0.625 |  | 0.86(0.74-0.99) | 0.040 |
| Unknown | 0.91(0.47-1.79) | 0.793 |  | 0.76(0.48-1.20) | 0.234 |
| PR status |  |  |  |  |  |
| Negative | Reference |  |  | Reference |  |
| Positive | 0.63(0.53-0.75) | <0.001 |  | 0.80(0.71-0.90) | 0.0003 |
| Unknown | 0.94(0.51-1.70) | 0.829 |  | 0.94(0.63-1.39) | 0.743 |
| HER2 status |  |  |  |  |  |
| Negative | Reference |  |  | Reference |  |
| Positive | 0.74(0.56-0.99) | 0.044 |  | 0.91(0.73-1.13) | 0.381 |
| Unknown | 0.71(0.56-0.89) | 0.004 |  | 0.91(0.76-1.08) | 0.275 |
| Surgery |  |  |  |  |  |
| Mastectomy | Reference |  |  | Reference |  |
| BCS | 0.67(0.55-0.80) | <0.001 |  | 0.78(0.68-0.90) | 0.001 |
| Radiotherapy |  |  |  |  |  |
| No/Unknown | Reference |  |  | Reference |  |
| Yes | 1.10(0.93-1.30) | 0.254 |  | 1.01(0.89-1.15) | 0.834 |
| Chemotherapy |  |  |  |  |  |
| No/Unknown | Reference |  |  | Reference |  |
| Yes | 1.01(0.87-1.18) | 0.860 |  | 0.76(0.69-0.85) | <0.001 |

**Abbreviation:** AJCC, American Joint Committee on Cancer; BCS, breast conserving surgery; BCSS, breast-cancer specific survival; CI, confidence interval; ER, estrogen receptor; HER2, human epidermal growth factor receptor-2; HR, hazard ratio; IDC, invasive ductal carcinoma; ILC, invasive lobular carcinoma; OS, overall survival; PR, progesterone receptor; PSM, propensity score matching.

**Supplemental table 6. Univariate cox analysis of BCSS and OS in breast-conserving patients with central and non-central tumor before PSM.**

| **Characteristic** | **BCSS** | |  | **OS** | |
| --- | --- | --- | --- | --- | --- |
|  | ***HR* (95%*CI*)** | **P value** |  | ***HR* (95%*CI*)** | **P value** |
| Year of diagnosis |  |  |  |  |  |
| 2004-2007 | Reference |  |  | Reference |  |
| 2008-2011 | 0.89(0.83-0.95) | 0.0006 |  | 0.99(0.94-1.03) | 0.563 |
| 2012-2015 | 0.80(0.75-0.86) | <0.001 |  | 0.95(0.90-0.99) | 0.032 |
| Age at diagnosis, yrs |  |  |  |  |  |
| 18-40 | Reference |  |  | Reference |  |
| 41-50 | 0.55(0.50-0.61) | <0.001 |  | 0.70(0.64-0.77) | <0.001 |
| 51-60 | 0.54(0.49-0.60) | <0.001 |  | 0.94(0.86-1.02) | 0.132 |
| 61-70 | 0.50(0.46-0.55) | <0.001 |  | 1.49(1.37-1.62) | <0.001 |
| Race |  |  |  |  |  |
| White | Reference |  |  | Reference |  |
| Black | 2.10(1.96-2.25) | <0.001 |  | 1.73(1.67-1.84) | <0.001 |
| Other | 0.85(0.76-0.94) | 0.003 |  | 0.76(0.70-0.81) | <0.001 |
| Unknown | 0.17(0.06-0.54) | 0.003 |  | 0.21(0.10-0.41) | <0.001 |
| Marital status |  |  |  |  |  |
| Unmarried | Reference |  |  | Reference |  |
| Married | 0.76(0.72-0.80) | <0.001 |  | 0.65(0.63-0.67) | <0.001 |
| Unknown | 0.86(0.74-1.01) | 0.067 |  | 0.82(0.74-0.91) | 0.0001 |
| Histological type |  |  |  |  |  |
| IDC | Reference |  |  | Reference |  |
| ILC | 0.72(0.64-0.82) | <0.001 |  | 0.91(0.84-0.98) | 0.012 |
| Others | 0.68(0.62-0.74) | <0.001 |  | 0.86(0.82-0.91) | <0.001 |
| Grade |  |  |  |  |  |
| G1 | Reference |  |  | Reference |  |
| G2 | 3.28(2.92-3.68) | <0.001 |  | 1.37(1.30-1.44) | <0.001 |
| G3/4 | 8.46(7.57-9.46) | <0.001 |  | 2.20(2.09-2.32) | <0.001 |
| Unknown | 2.98(2.44-3.64) | <0.001 |  | 1.37(1.23-1.53) | <0.001 |
| Tumor stage, AJCC 6th |  |  |  |  |  |
| T1 | Reference |  |  | Reference |  |
| T2 | 3.68(3.49-3.89) | <0.001 |  | 2.03(1.96-2.11) | <0.001 |
| Node stage, AJCC 6th |  |  |  |  |  |
| N0 | Reference |  |  | Reference |  |
| N1 | 2.75(2.59-2.92) | <0.001 |  | 1.62(1.56-1.70) | <0.001 |
| N2 | 6.45(5.91-7.04) | <0.001 |  | 3.05(2.84-3.27) | <0.001 |
| N3 | 11.59(10.36-12.96) | <0.001 |  | 5.11(4.65-5.63) | <0.001 |
| ER status |  |  |  |  |  |
| Negative | Reference |  |  | Reference |  |
| Positive | 0.33(0.31-0.35) | <0.001 |  | 0.55(0.53-0.58) | <0.001 |
| Unknown | 0.48(0.40-0.58) | <0.001 |  | 0.73(0.64-0.83) | <0.001 |
| PR status |  |  |  |  |  |
| Negative | Reference |  |  | Reference |  |
| Positive | 0.36(0.34-0.38) | <0.001 |  | 0.59(0.57-0.61) | <0.001 |
| Unknown | 0.52(0.44-0.62) | <0.001 |  | 0.75(0.67-0.83) | <0.001 |
| HER2 status |  |  |  |  |  |
| Negative | Reference |  |  | Reference |  |
| Positive | 1.07(0.92-1.23) | <0.038 |  | 0.95(0.86-1.06) | 0.345 |
| Unknown | 1.17(1.10-1.25) | <0.001 |  | 1.03(0.98-1.08) | 0.242 |
| Chemotherapy |  |  |  |  |  |
| No/Unknown | Reference |  |  | Reference |  |
| Yes | 3.78(3.55-4.02) | <0.001 |  | 1.47(1.42-1.52) | <0.001 |
| Site |  |  |  |  |  |
| Non-central | Reference |  |  | Reference |  |
| Central | 0.90(0.80-1.02) | 0.102 |  | 1.10(1.02-1.19) | 0.0173 |

**Abbreviation:** AJCC, American Joint Committee on Cancer; BCS, breast conserving surgery; BCSS, breast-cancer specific survival; CI, confidence interval; ER, estrogen receptor; HER2, human epidermal growth factor receptor-2; HR, hazard ratio; IDC, invasive ductal carcinoma; ILC, invasive lobular carcinoma; OS, overall survival; PR, progesterone receptor; PSM, propensity score matching.

**Supplemental table 7. Multivariable Cox analysis for BCSS and OS in patients received breast-conserving treatment before PSM.**

| **Characteristic** | **BCSS** | |  | **OS** | |
| --- | --- | --- | --- | --- | --- |
|  | ***HR* (95%*CI*)** | ***P* value** |  | ***HR* (95%*CI*)** | ***P* value** |
| Year of diagnosis |  |  |  |  |  |
| 2004-2007 | Reference |  |  | Reference |  |
| 2008-2011 | 0.97(0.90-1.04) | 0.359 |  | 0.97(0.93-1.02) | 0.317 |
| 2012-2015 | 0.92(0.81-1.04) | 0.187 |  | 0.91(0.84-0.99) | 0.035 |
| Age at diagnosis, yrs |  |  |  |  |  |
| 18-40 | Reference |  |  | Reference |  |
| 41-50 | 0.83(0.75-0.92) | 0.0003 |  | 0.90(0.82-0.99) | 0.023 |
| 51-60 | 0.93(0.84-1.02) | 0.116 |  | 1.28(1.17-1.39) | <0.001 |
| 61-70 | 1.07(0.97-1.18) | 0.193 |  | 2.21(2.03-2.40) | <0.001 |
| Race |  |  |  |  |  |
| White | Reference |  |  | Reference |  |
| Black | 1.37(1.27-1.47) | <0.001 |  | 1.41(1.341.49) | <0.001 |
| Other | 0.82(0.73-0.91) | 0.0003 |  | 0.81(0.75-0.87) | <0.001 |
| Unknown | 0.18(0.06-0.56) | 0.003 |  | 0.21(0.10-0.42) | <0.001 |
| Marital status |  |  |  |  |  |
| Unmarried | Reference |  |  | Reference |  |
| Married | 0.85(0.80-0.90) | <0.001 |  | 0.72(0.69-0.74) | <0.001 |
| Unknown | 0.95(0.81-1.11) | 0.543 |  | 0.87(0.78-0.97) | 0.010 |
| Histological type |  |  |  |  |  |
| IDC | Reference |  |  | Reference |  |
| ILC | 1.09(0.96-1.25) | 0.181 |  | 0.97(0.90-1.06) | 0.604 |
| Others | 0.88(0.81-0.96) | 0.004 |  | 0.94(0.90-0.99) | 0.032 |
| Grade |  |  |  |  |  |
| G1 | Reference |  |  | Reference |  |
| G2 | 2.34(2.08-2.64) | <0.001 |  | 1.21(1.15-1.28) | <0.001 |
| G3/4 | 3.61(3.19-4.08) | <0.001 |  | 1.59(1.50-1.69) | <0.001 |
| Unknown | 2.16(1.76-2.64) | <0.001 |  | 1.23(1.10-1.37) | 0.0002 |
| Tumor stage, AJCC 6th |  |  |  |  |  |
| T1 | Reference |  |  | Reference |  |
| T2 | 1.90(1.79-2.02) | <0.001 |  | 1.61(1.54-1.67) | <0.001 |
| Node stage, AJCC 6th |  |  |  |  |  |
| N0 | Reference |  |  | Reference |  |
| N1 | 1.96(1.83-2.09) | <0.001 |  | 1.54(1.47-1.61) | <0.001 |
| N2 | 3.74(3.41-4.10) | <0.001 |  | 2.62(2.43-2.83) | <0.001 |
| N3 | 5.79(5.15-6.50) | <0.001 |  | 2.94(3.57-4.36) | <0.001 |
| ER status |  |  |  |  |  |
| Negative | Reference |  |  | Reference |  |
| Positive | 0.82(0.75-0.89) | <0.001 |  | 0.81(0.76-0.86) | <0.001 |
| Unknown | 0.86(0.62-1.17) | 0.332 |  | 0.96(0.78-1.17) | 0.680 |
| PR status |  |  |  |  |  |
| Negative | Reference |  |  | Reference |  |
| Positive | 0.68(0.63-0.74) | <0.001 |  | 0.83(0.78-0.87) | <0.001 |
| Unknown | 0.93(0.71-1.22) | 0.588 |  | 0.92(0.77-1.09) | 0.316 |
| HER2 status |  |  |  |  |  |
| Negative | Reference |  |  | Reference |  |
| Positive | 0.59(0.51-0.69) | <0.001 |  | 0.77(0.69-0.85) | <0.001 |
| Unknown | 0.94(0.84-1.05) | 0.260 |  | 0.96(0.87-1.03) | 0.230 |
| Chemotherapy |  |  |  |  |  |
| No/Unknown | Reference |  |  | Reference |  |
| Yes | 1.28(1.19-1.38) | <0.001 |  | 0.89(0.85-0.93) | <0.001 |
| Tumor location |  |  |  |  |  |
| Non-central | Reference |  |  | Reference |  |
| Central | 0.99(0.87-1.12) | 0.850 |  | 1.09(1.01-1.18) | 0.030 |

**Abbreviation:** AJCC, American Joint Committee on Cancer; BCS, breast conserving surgery; BCSS, breast-cancer specific survival; CI, confidence interval; ER, estrogen receptor; HER2, human epidermal growth factor receptor-2; HR, hazard ratio; IDC, invasive ductal carcinoma; ILC, invasive lobular carcinoma; OS, overall survival; PR, progesterone receptor; PSM, propensity score matching.
